# Low-Grade Glioma Segmentation Based on CNN with Fully Connected CRF

**DOI:** 10.1155/2017/9283480

**Published:** 2017-06-13

**Authors:** Zeju Li, Yuanyuan Wang, Jinhua Yu, Zhifeng Shi, Yi Guo, Liang Chen, Ying Mao

**Affiliations:** ^1^Department of Electronic Engineering, Fudan University, Shanghai, China; ^2^Key Laboratory of Medical Imaging Computing and Computer Assisted Intervention of Shanghai, Shanghai, China; ^3^Department of Neurosurgery, Huashan Hospital, Fudan University, Shanghai, China

## Abstract

This work proposed a novel automatic three-dimensional (3D) magnetic resonance imaging (MRI) segmentation method which would be widely used in the clinical diagnosis of the most common and aggressive brain tumor, namely, glioma. The method combined a multipathway convolutional neural network (CNN) and fully connected conditional random field (CRF). Firstly, 3D information was introduced into the CNN which makes more accurate recognition of glioma with low contrast. Then, fully connected CRF was added as a postprocessing step which purposed more delicate delineation of glioma boundary. The method was applied to T2flair MRI images of 160 low-grade glioma patients. With 59 cases of data training and manual segmentation as the ground truth, the Dice similarity coefficient (DSC) of our method was 0.85 for the test set of 101 MRI images. The results of our method were better than those of another state-of-the-art CNN method, which gained the DSC of 0.76 for the same dataset. It proved that our method could produce better results for the segmentation of low-grade gliomas.

## 1. Introduction

Among all brain tumors, glioma is the most severe [1]. According to the World Health Organization (WHO) criteria [2], gliomas were categorized into four grades from level I to level IV according to tumor malignancy. Normally, grade III and grade IV gliomas are called as high-grade gliomas (HGG) and grade I and grade II as low-grade gliomas (LGG). LGG could be further classified into astrocytomas, oligodendroglioma, and oligodendrocytes astrocytoma based on pathological type.

Magnetic resonance imaging (MRI) is the most common imaging diagnostic technique in the clinical diagnosis of gliomas. With MRI images, the accurate segmentation of gliomas is one of the most crucial procedures in treatment planning and follow-up evaluations. However, manual labeling is very time-consuming, and it is difficult to adopt a unified standard for segmentation. Meanwhile, automatic segmentation is still hard to be achieved because of the diversity of gliomas in size, shape, and location [3]. Several limits of medical images such as the intensity inhomogeneity and unexpected intensity ranges of tissues would also cause difficulty for automatic segmentation of glioma [4].

A large number of algorithms have been developed to complete the task of tumor segmentation. Many traditional segmentation methods were based on gray scale values, such as fuzzy clustering and region growing [5]. These methods would be likely to fail when processing nonenhanced tumor images. Another kind of popular methods was multiatlas segmentation, which was based on the correlation of the priori brain atlas and the medical images to be processed [6]. However, these methods are often problematic when the atlases and target images are obtained via different imaging protocols and the deformable registration is also considered as a difficult process.

Recently, several methods related with machine learning have been applied in brain tumor segmentation. Parisot et al. used the prior knowledge to classify the tumor first and then used another graph to determine the class of each voxel [7]. Huang et al. utilized the sparseness of samples to build up a particular dictionary and used a softmax model to optimize the error reconstruction coefficients for different classes [8]. Random forests have been considered to be good at dealing with a great number of features to accomplish brain tumor segmentation. Meier et al. applied a set of dedicated features to get decision forests to discriminate pathological regions from brain MRI volumes [9]. In addition, Markov random field (MRF) and conditional random field (CRF) are also often mentioned to obtain smooth edges. Zhao et al. proposed a semisegmentation method based on the MRF [10], in which one slice was labeled and other slices were sequentially labeled based on a MRF label. Meier et al. estimated the CRF to improve the voxel-wise classification performance on the top of the decision forest classifier [11]. These conventional machine learning methods are often based on a large number of features extracted from the image, reflecting the shape, gray value, and texture of the tumor area. But an important problem with these approaches is that the computation of too many features is too time-consuming and particular feature can cause difficulties in tuning.

Another kind of approach to segment gliomas is based on the well-known convolutional neural network (CNN). Primarily due to its abilities to obtain image global and local information directly from the convolution kernels, CNNs have made breakthrough progress in image processing and object recognition and been wildly used thereafter [12]. CNNs have shown good performances in the field of medical image processing in recent years, not only in terms of accuracy, but also in terms of efficiency [13]. Pereira et al. developed two CNN structures with different depths to deal with the HGG and the LGG [14]. Dvorak et al. evaluated the effectiveness of different patch selection strategies based on the segmentation results of CNNs [15]. Havaei et al. proposed a multiscale CNN structures in order to make better use of local and global information [16]. Rao et al. combined random forests with the final output of CNNs to achieve better classification results [17]. Several CNN methods mentioned previously are based on a two-dimensional convolution kernels and do not make good use of the natural three-dimensional (3D) information of medical images. Typically, 3D filters can take fully advantage of 3D connection characteristics of images. Kamnitsas et al. [18] evaluated the use of 3D filters. However, the 3D convolution algorithm limits the size of convolution kernels and causes a great increase of the computation load. Furthermore, 3D filters require high resolution on the vertical plane, while actual MRI images usually need interpolation and do not have such high resolution. The process of interpolation and down sampling in 3D filters often brings additional errors in segmentation. Therefore, how to make good use of 3D information with CNN in gliomas segmentation still remains an important problem.

On the other hand, segmentation methods mentioned above mainly focused on the segmentation of the HGG. Although the internal structure of the LGG is simpler than that of the HGG, the segmentation of the LGG is considered more difficult because of its lower contrast and smaller size [14]. Thus, these segmentation methods mentioned above often do not produce good results when dealing with the LGG.

As it is known, the lesion area of the LGG is more distinguishable from T2flair MRI than from other MRI modalities [19]. So in this study, we chose T2flair modal MRI images as the original data for image processing. LGG has high signal in the T2flair images. Compared with HGG, the signal intensity distribution of LGG is more uniform and the boundaries of tumors and surrounding brain tissues tend to be clearer. In addition, LGG usually shows no necrosis, perifocal edema, or hemorrhagic foci. Oligodendroglioma and astrocytoma were two major types of LGG. The two subtypes could be distinguished radiologically by the presence of calcification. Generally, calcification inside oligo tumor turns out hypointensity on T2-weighted and isointensity on T1-weighted precontrast MRI.

In this paper, a new method is presented aiming at automatic segmentation of LGG MRI images. Main contributions of the paper are as follows. Firstly, the effect of different CNN depths and the number of neurons in the fully connected layers on the segmentation result were thoroughly evaluated. Secondly, in order to use the 3D information, nearby slices were set into the network and connected with a fully connected CRF. Lastly, the results on the LGG T2flair dataset showed that the method is better than the state-of-the-art CNN method.

The rest of the paper is organized as follows: in Section 2, we present our materials and method flows. Experimental design, results, and discussion can be found in Section 3. Finally, the main conclusions are presented in Section 4.

## 2. Materials and Methods

### 2.1. Patients

All data from patients used in our study were obtained from Shanghai Huashan Hospital. These patients were diagnosed between July 2013 and March 2016, and MRI images of these patients were collected at the time of diagnosis without any treatment. All 160 cases of data are described in detail in Table 1. We randomly selected 59 of them as the training data and 101 as the test data. Manual labeling of the tumor area was performed by two experienced neurosurgeons.

The size of the MRI images was 512 × 400. MRI images were stored as 16-bit unsigned integer. All images were acquired according to the following parameters: pixel width = 0.47 mm, slice spacing = 2 mm, repetition time = 9000 ms, echo time = 99 ms, inversion time = 2501 ms, and flip angle = 150°.

### 2.2. Data Analysis

The original MRI data contains a lot of information which is not related to the segmentation problem, and these noises will greatly affect the tumor segmentation [3]. Some pretreatment was firstly operated to get rid of these noises.

BrainSuite is an open-source software and is able to automatically process the human brain medical images [20]. BrainSuite was utilized to remove the skull and scalp from MRI images and corrected nonuniformity problem of images.

### 2.3. CNNs for Tumor Segmentation

**Figure 1:**
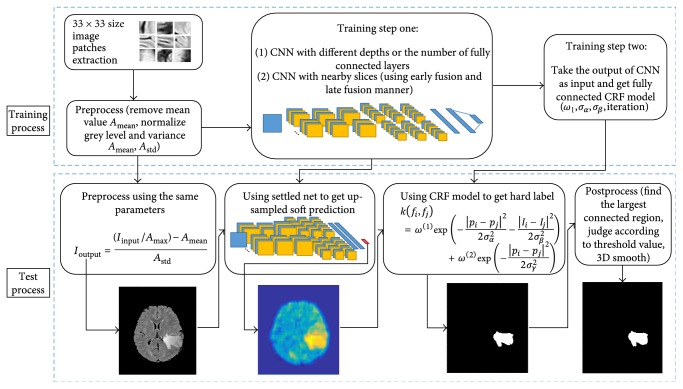
The flow chart of our method.

Figure 1 presents the overview of our approach, which is divided into several parts. The proposed method was also demonstrated in Algorithm 1. The further explanation will be given in the following sections.

#### 2.3.1. The Preparation of the Input

The major idea of using CNN to segment gliomas is to take tumor segmentation as the problem of tumor recognition. Because the proportion of tumors in the brain is very small, some normal CNN network structures of image recognition could not get good segmentation results of gliomas [21]. Instead of utilizing the full-sized images in the training phase, CNN was trained using patches randomly extracted from the images in this study. The conventional processing is to divide images into several patches during the training and set categories of center points as targets. The method was widely used in the medical image processing with unbalanced data [22]. More storage and more time were required by the training strategy using patches compared with training strategy using full-sized images. However, the former one is more suitable for tumor segmentation because the portion of the tumor regions is very small and the strategy using full-sized images would cause false positive results with unbalanced samples. The tumor region could be sampled more with the strategy using patches by picking more samples in the tumor regions on purpose.

In our study, we divided MRI images into 33 pixels × 33 pixels size patches randomly at the training stage. Normally, about 50 patches were extracted from a single slice. The training set contained about 230,000 patches from 59 patients' brain images.

Taking into account the uneven distribution of image data, we chose an unbalanced selection method to obtain sufficient tumor samples. There were about 40% of samples in the channel containing tumors.

After the completion of sample extraction, we adopted several preprocessing methods. We removed the mean grey level of patches and normalized gray value and variance. It is worth being noted that these parameters are preserved for the test image to do the same processing.

#### 2.3.2. CNN Base Line Structure

We selected one of the most advanced CNN structures as the base line. The selected CNN took part in the Brain Tumor Segmentation Challenge 2013 (BRATS 2013) and ranked first place in BRATS 2013 and second place in BRATS 2015 [14]. The network consisted of seven convolution layers, and structures are briefly described as the base line in Table 2. The network utilized small and continuous convolution kernels to enhance tumor recognition ability of network without increasing computation.

Every convolution layer was followed by active layers, and the dropout layer was set after each fully connected layer. According to regular CNN strategy, we chose rectifier linear units (ReLU) as the function of active layers.

It should be mentioned that we put the entire image into the network in the phase of testing to reduce the processing time. For the purpose of getting the correct and accurate labeling map, we removed the dropout and softmax loss layers of the network in testing.

#### 2.3.3. Building Deeper Networks

Some research results on the CNN showed that objects would be recognized better with increasing depth of the network structure and the number of neurons in the fully connected layers. Therefore, we conducted a number of contrast experiments with different depths and different number of neurons in the fully connected layers based on the base line. We referred to a well-behaved network structure at the time of designing the network structure [23]. Detailed information is also shown in Table 2.

#### 2.3.4. Adding Near Slices into Networks

**Figure 2:**
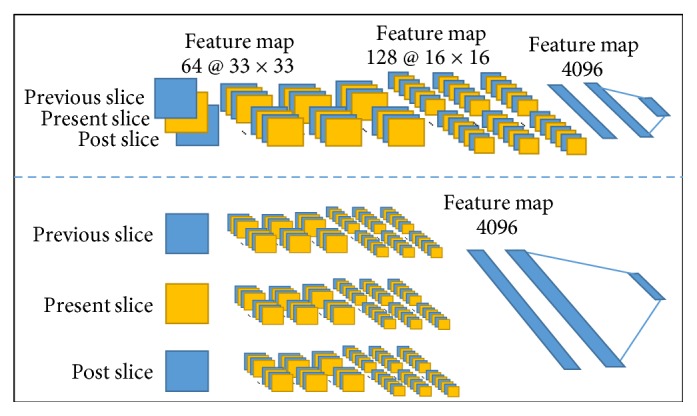
Two network structures which can make better use of 3D information. The implementation structure of early fusion is shown above, and the figure below is the diagram of late fusion.

As previously mentioned, it is a tricky problem to input the 3D information into the network without increasing the computational load and bringing the complex registration process. This problem cannot be well solved by existing methods. In order to drop out a solution, two ideas in motion recognition from video processing were introduced into our CNN structure. Two new network architectures were shown in Figure 2, called early fusion and late fusion processes.

In the field of video processing, Karpathy et al. reported that early fusion structure obtained more accurate local motion recognition and late fusion compared the contents of each channel to obtain the better recognition of global motion [24]. Video processing and 3D brain MRI images have similar characteristics; although several frames nearby are not exactly the same, relative information could provide assistance to make more specific judgments. In fact, neighbor slices were always referred to get better differentiation of the tumor area at the time of manual labeling. Based on this fact, introducing these structures of combining 3D information could be helpful in segmenting tumor regions.

It is worth being mentioned that we utilized the network with a deeper structure and more neurons in the fully connected layers as a basis. The basic structure is referred to as “Base line + deeper + more fc” CNN structure in Table 2. As seen in the figure, early fusion mixes the information of three slices together at the beginning of the network and three relative slices shared the same network structure parameters. Each slice was utilized to train a one-way network in the structure of late fusion and finally connected together by the fully connected layers.

The connection of different slices in the network has many advantages. Firstly, introducing 3D information can make the network identify the tumor region more accurately. This may be of benefit to the CRF decision. Secondly, it is possible to effectively avoid missegmentation for similar tumor regions in other brain regions. Combining nearby slices can correct erroneous identification by the use of 3D information. Moreover, the structures combined with 3D information could help CNN recognize the upper and lower surfaces of the tumor better. The accurate identification of tumor regions would also bring benefits to the segmentation of small tumors.

#### 2.3.5. Fully Connected CRF for Further Identification

Current CNN structures for the tumor segmentation [14–17] have several limitations due to the structure. First of all, the receptive field that corresponds to a single neuron of the last fully connected layer is too large. Take our network structure for example, due to the presence of two pooling layers, one neuron corresponds to four pixels in images. What is more, the CNN was limited by the lack of space and edge constraints compared with other machine learning methods. So the result of the segmentation is rough on edges. Secondly, for MRI images, the contrast and the gray scale of images from different patients are different. In addition, the contrast of the LGG is often lower than that of the HGG, increasing the difficulty for accurate identification of whole tumors. Because of these reasons, sometimes the CNN can only recognize a part of the tumor area and sometimes the region near the tumor is misidentified.

A possible improvement of the CNN is to integrate the CRF into the network structure to refine the segmentation results [25]. CRF is a framework for building probabilistic models. Iterative parameter estimation algorithms were always applied for CRF [26]. Krähenbühl reduced the complexity of fully connected CRF computation from quadratic complexity to linear complexity through an optimization algorithm based on the mean field approximation [27]. On his basis, we used the network features as the contribution of each category and optimized the probability model in a wide domain. The energy function that we used is

(1) $$\begin{array}{c} E\left(x\right)=\underset{i}{\sum }\theta_{u}\left(x_{i}\right)+\underset{i,j,i\neq j}{\sum }\theta_{p}\left(x_{i}+x_{j}\right), \end{array}$$

where *θ*_*u*_(*x*_*i*_) is the unary potential which is computed independently for each pixel. In our study, we utilized features extracted from the CNN to calculate this parameter.

(2) $$\begin{array}{c} \theta_{u}\left(x_{i}\right)=-\log P\left(x_{i}\right), \end{array}$$

where *P*(*x*_*i*_) is the label assignment probability. We set the output of the last fully connected layer of the CNN as *P*(*x*_*i*_). It should be noted that, the bi-cubic method was used to interpolate the score map since the CNN output size is four times smaller than the image. The other parts of the energy function are the pairwise potentials, which reflect the relationship between any two pixels. Pairwise potentials were defined as

(3) $$\begin{array}{c} \theta_{p}\left(x_{i}+x_{j}\right)=\mu_{p}\left(x_{i}+x_{j}\right)\overset{K}{\underset{m=1}{\sum }}\omega^{\left(m\right)}k^{\left(m\right)}\left(f_{i},f_{j}\right), \end{array}$$

where *μ*_*p*_(*x*_*i*_ + *x*_*j*_) is a function that determines whether a point is the same or not. The Gaussian kernel *k*^(*m*)^ was calculated as follows:

(4) $$\begin{array}{c} k\left(f_{i},f_{j}\right)=\omega^{\left(1\right)}\exp\left(-\frac{{\left|p_{i}-p_{j}\right|}^{2}}{2\sigma_{\alpha }^{2}}-\frac{{\left|I_{i}-I_{j}\right|}^{2}}{2\sigma_{\beta }^{2}}\right)+\omega^{\left(2\right)}\exp\left(-\frac{{\left|p_{i}-p_{j}\right|}^{2}}{2\sigma_{\gamma }^{2}}\right), \end{array}$$

where *p*_*i*_ and *I*_*i*_ represent the location and intensity of the corresponding pixel *i*, respectively. As seen from the formula, the first Gaussian kernel is related to the location and intensity of a particular pixel, which is the main convergence factor of the graph model. However, the second kernel is only related to the location of the pixel, which makes the graph model smoother. In our experiments, the iterations of the CRF algorithm also had a great influence on the segmentation result. These parameters of the CRF should be obtained through the second training phase of the training set.

#### 2.3.6. Postprocessing

After obtaining the segmentation results of the CRF output, the segmentation results were corrected using several morphology methods. We chose the largest connectivity region of each slice as the candidate region. Then regions containing the area greater than 300 pixels were selected as identified tumors. After yielding the segmentation results for each slice, 3D smoothing of the tumor was performed by box filter with 3-dimensional convolution kernel to make segmentation results more natural.

## 3. Results and Discussion

### 3.1. Experimental Setup and Evaluation

All of our experiments were built on top of MatConvNet [28], a MATLAB toolbox implementing CNNs for computer vision applications.

In order to make experimental results more contrastive, we used the same parameters when models were trained. Network parameters were initialized by using the improved Xavier method [29], and the echo setting for training was 25. The initial learning rate was 3 × 10^−3^ and declined to the final 3 × 10^−5^ using the logarithmic descent method.

In the process of training the fully connected CRF parameters, we used a relatively simple genetic algorithm to specify the optimal parameters. As shown in Table 3, all the parameters were discretized. *ω*_1_ and *σ*_*γ*_ were considered unimportant and were set to 5 and 5, separately. At first, we generated a set of parameters randomly and then changed four parameters one by one in order to optimize the selection, retaining the best one. Thus, 27 cycles were required to estimate the parameters of the fully connected CRF model. The parameters corresponding to the best overall segmentation results of the 59 training data were chosen as the optimized parameters during the second training phase.

In order to evaluate the accuracy of the segmentation results, we used three indices as usual [3] Dice similarity coefficient (DSC) [30], positive predictive value (PPV), and sensitivity. The DSC evaluated the segmentation results globally by calculating the overlapping parts, which often serve as an indicator of the overall outcome of segmentation results.

(5) $$\begin{array}{c} DSC=\frac{2TP}{FP+2TP+FN}, \end{array}$$

where TP, FP, and FN represent the regions of the true positive, the false positive, and the false negative, respectively. The PPV calculated the accuracy of the segmentation result. The higher the coefficient is, the less the nontumor area can be covered by the segmentation results.

(6) $$\begin{array}{c} PPV=\frac{TP}{TP+FP}. \end{array}$$

The sensitivity reflects the sensitivity of the algorithm to the tumor area. The higher the sensitivity, the more regions of the tumor that are included in the segmentation results.

(7) $$\begin{array}{c} Sensitivity=\frac{TP}{TP+FN}.\; \end{array}$$

### 3.2. Results for Different Network Depths

**Figure 3:**
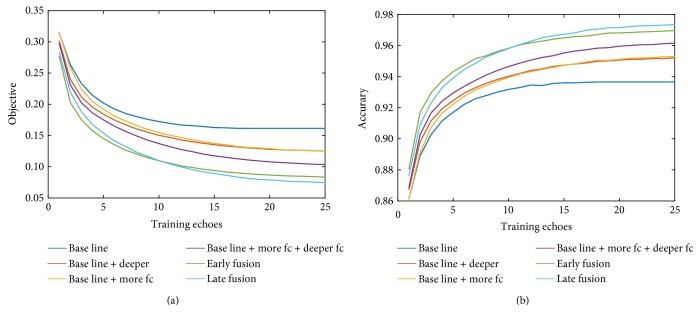
The training parameters of different network structures. (a) shows the convergence of the objective function during training, and (b) shows the error change during training.

Four experiments were designed with different CNN depths and architectures. The design of the network was mentioned in Table 2. The convergence of the objective function and the error rate during the training is shown in Figure 3.

We can see from the figure that increasing the depth of the network and the number of neurons in the fully connected layers can make the network stabilize more quickly and gain better results. Moreover, deepening the network structure and increasing the number of neurons in the fully connected layers at the same time could further enhance the training results.

The segmentation results of training set and test set were evaluated, as shown in Tables 4 and 5. Quantitative results showed that making network deeper can improve the segmentation results without significantly increasing network parameters. When the network is deep enough, the increase of the number of neurons in the fully connected layers would also improve the tumor recognition ability of the network.

**Figure 4:**
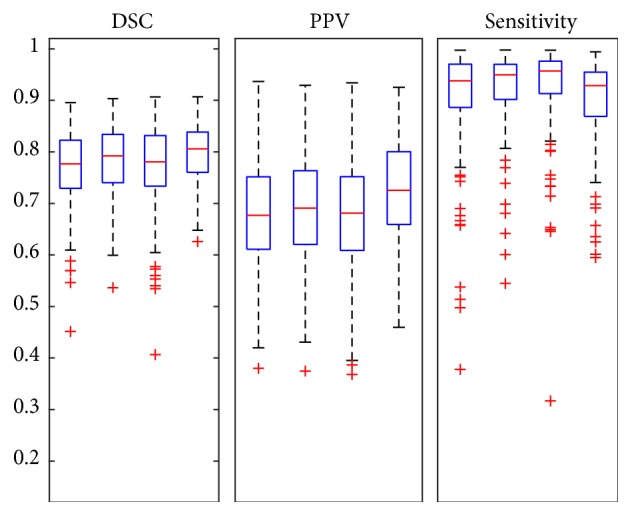
Boxplots of all data segmentation results of different CNN structures. The first of each group represents the base line, the second represents the deeper structure of the network, the third represents the network with more neurons in the fully connected layers, and the last one is the network with both deeper and more neurons in the fully connected layers.

All 160 cases of tumor segmentation results were presented in Figure 4. It can be seen that the tumor recognition ability of complex network was better than the ones with simple network structure. Nevertheless, the improved network can also better handle some special cases.

**Figure 5:**
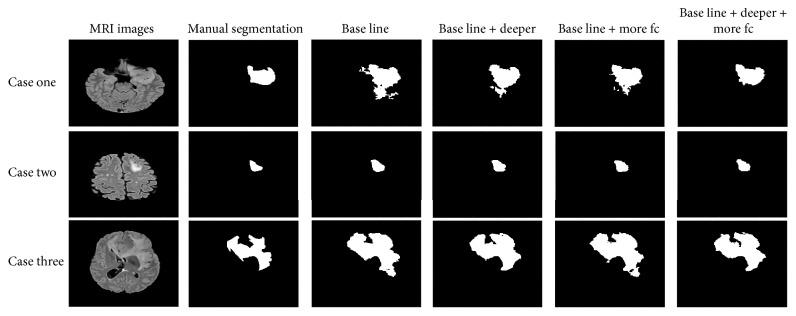
Segmentation results for networks with different depths. Each row corresponds to a case, and each column corresponds to a network structure of the segmentation results.

The effect of changing network structrue on the segmentation results can be better reflected in Figure 5(). We showed three examples of segmentation in Figure 5(), and it can be seen that the segmentation results became more detailed and accurate when the network was deepened or the number of neurons in the fully connected layers was increased.

The results were also consistent with the conclusion in the previous quantitative analysis. The promotion of the PPV was the most remarkable, and the nontumor region covered by segmentation results was decreased.

**Figure 6:**
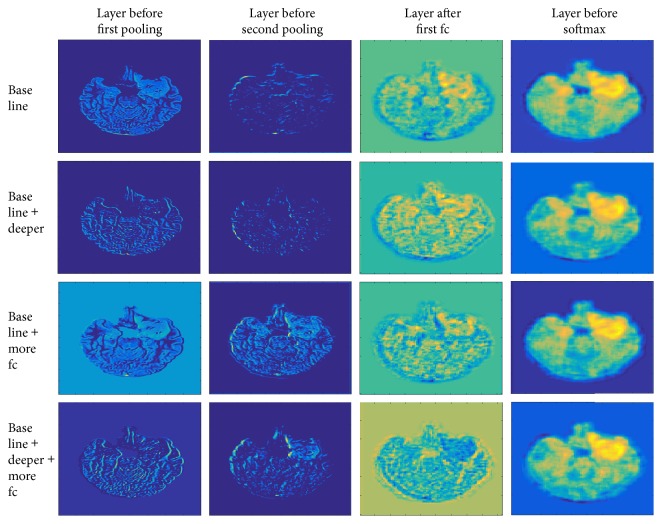
Features in networks of case one in Figure 4. Each column corresponds to a network structure, and each row corresponds to one kind of depth; the output of the last filter in the filter bank was selected.

We also looked into the difference in the network architecture by analyzing network features values. Representative features in the network from different depths were presented in Figure 6(). As some of the research in computer vision reported [31], the lower layers represent the information such as edges, contours, and intensities. With the deepening of the network, the characteristics become more different with the input images. Besides, the regions from different classes become more and more obvious, especially after the fully connected layers. It can be seen that networks with more neurons in the fully connected layers can better identify tumor regions at the same depth. Because the increase of neurons in the fully connected layers gives more choices of fitting, the lower layers have the opportunity to establish better parameters.

The effect of increasing the depth of the network can be seen from the diagram in the last column. With the deepening of the network, image recognition is significantly more detailed and the resolution of pixel recognition is higher. Fine identification of images can benefit the reduction of recognition errors. This may be because the increase in the number of convolution layers leads to multiple processing of the same pixel point.

By doing so, the CNN could effectively reduce the adverse impact of surrounding pixels so that recognition is more precise.

### 3.3. Results for Adding the Near Slices and Fully Connected CRF

Similarly, we presented the training parameters of the network structure after adding the neighboring slice information in Figure 3. By comparison, it can be seen from Figure 3 that the parameters of the training after the addition of 3D information yield a much better convergence result. This was because the increased 3D information improves the recognition ability of the network. However, maybe due to the noises brought by 3D information, the actual segmentation results were not very satisfactory.

We also showed the result of the segmentation of the training set and the test set in Tables 6 and 7, after combined with the fully connected CRF. It can be seen that the combination of the CNN and the CRF can improve the recognition effect of tumors to a great extent. More importantly, the combination of these two 3D structures with the CRF seems to achieve better results. The results of early fusion had a higher DSC, and the results of late fusion were able to achieve higher sensitivity with similar global segmentation results.

**Figure 7:**
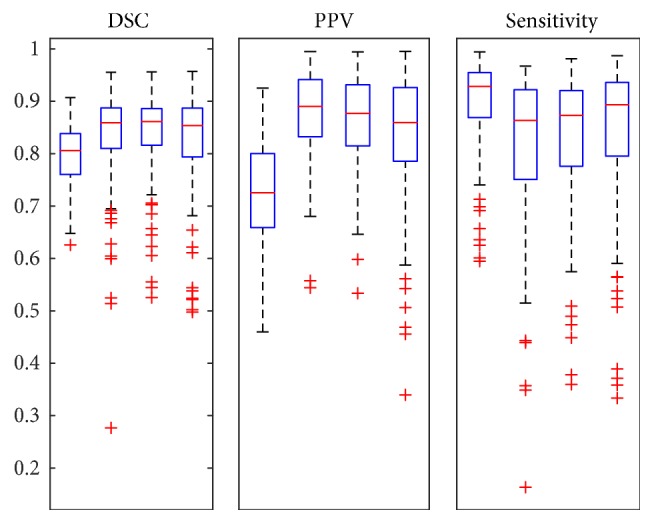
Boxplots of all data segmentation results of different CNN structures combined with fully connected CRF. The first plots of each group represent results of one way CNN, the second plots represent results of one way CNN connected to CRF, the third plots represent results of the early fusion structure with CRF, and the last ones are the results of the late fusion structure with CRF.

The results are more evident in the globally validated Figure 7. Compared with the single way CNN, the combination of the CNN and the CRF achieved a significant improvement. Moreover, early fusion and late fusion accessed to more promotion in different aspects.

**Figure 8:**
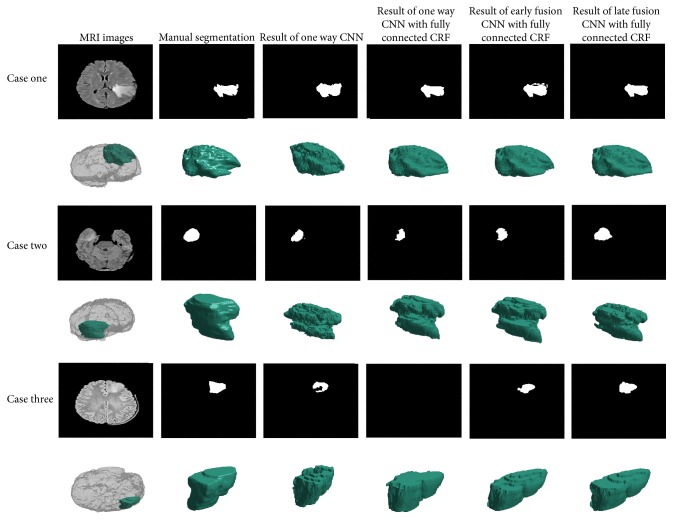
Segmentation results of different network structures combined with the fully connected CRF. Each row corresponds to a case, and each column corresponds to a network structure of the segmentation results. The 3D reconstructions of tumors are shown in the second row of each group.

In Figure 8(), we showed the superiority of our method in three examples. In the first case, we can see that the contours of the tumor obtained with one way CNN are very rough and the boundaries acquired are not very good. The segmentation results are more precise and more accurate after combining the CNN with the fully connected CRF. It is worth being mentioned that we did not use the fully connected CRF to smoothen the edges completely. We found that appropriate iteration time of CRF would lead to better segmentation than the one with fully smoothing. Therefore, the segmentation of our method might be rugged and preserved good edges of the tumor regions.

In the second case and the third case, we showed the benefits of combining nearby slices. The CNN combined with 3D information can identify the tumor area globally. In the second case, two network structures combined with 3D information can better identify the whole tumor area, even if a part of the tumor had very poor contrast.

In the third case, the upper surface of the tumor cannot be recognized by the single way CNN. But two kinds of network structures combined with 3D information can complete segmentation successfully and make the tumor identification more detailed.

As for two structures of early fusion and late fusion, we can see that the results of the former are more detailed and the latter are more likely to recognize more regions. Three convolution channels combined in late fusion showed responses to larger regions. This may be due to the fact that separate convolution channels obtained more 3D information but were more susceptible to noises.

**Figure 9:**
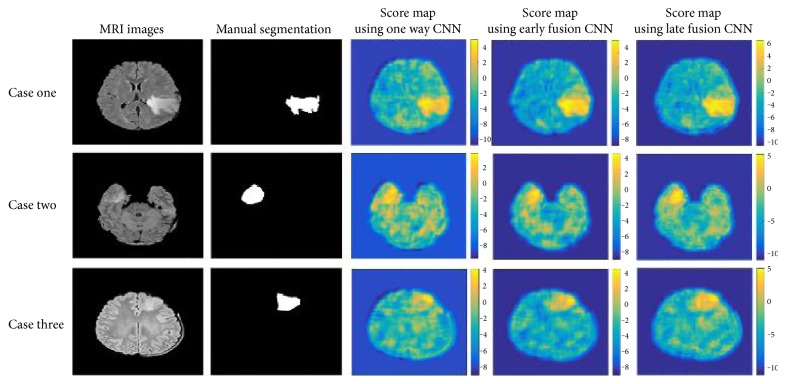
The score maps that are entered into the CRF of different network structures. Each row corresponds to a case and each column corresponds to a network structure.

We can further explore the advantages of our method of combining 3D information with Figure 9. In Figure 9, we showed the unary potential preparing for the input of the fully connected CRF. These features were set as the output of the last fully connected layers in CNNs. The color bars next to the score map showed the intensity distribution in the graph.

It can be seen that the intensity distribution of the unary potential obtained from two CNN structures with 3D information was more extensive and the color of the tumor region shown in the figure was brighter. The results corresponded to the point we said previously that the CNN can recognize tumor regions more certainly after adding 3D information. This capability would be beneficial for better recognition of the fully connected CRF, as can be inferred from (1).

### 3.4. Operation Times

Thanks to the CNN processing speed advantage and the simplified calculation method of the fully connected CRF, our proposed pipeline can be completed in a very short time. Specifically, by using GPU NVIDIA Quadro 600 on an Intel Xeon E5620 2.40 GHz machine, we need one to seven days to train networks, and the specific time depends on the network complexity. After the network is obtained, the segmentation time was much less since the segmentation process required only the forward operation of the network. A slice takes only 2 to 10 seconds to get the segmentation results, and all images of a patient need 2 to 10 minutes. Similarly, the specific time varies according to the network structure. Compared with the traditional machine learning methods, our method is very advantageous in computing time [3].

## 4. Conclusions

In this study, we explore better ways to segment the LGG based on the CNN in the binary framework. We firstly studied the effect of different depths and the number of neurons in the fully connected layers on the tumor segmentation. It was found that the deepening of the network can optimize the segmentation results without increasing the amount of computation. Increasing the number of neurons in the fully connected layers under the same conditions can also improve the segmentation results. Next, we found that the use of the fully connected CRF as the CNN postprocessing can improve the segmentation results of the contour and edge and greatly enhances the segmentation results. At last, we proposed two kinds of network structures combined with 3D information. Experiments showed that the combination of these two structures and CRF can get better results. Early fusion can improve the segmentation results globally, and late fusion can make the segmentation results more sensitive. Using our proposed workflow, we can achieve better results on the LGG segmentation than one of the best CNN tumor segmentation methods at present. In order to better verify the applicability of our method, we will have a more detailed identification of brain tumors and validate our method on BRATS database in the future.

## Figures and Tables

**Algorithm 1:**
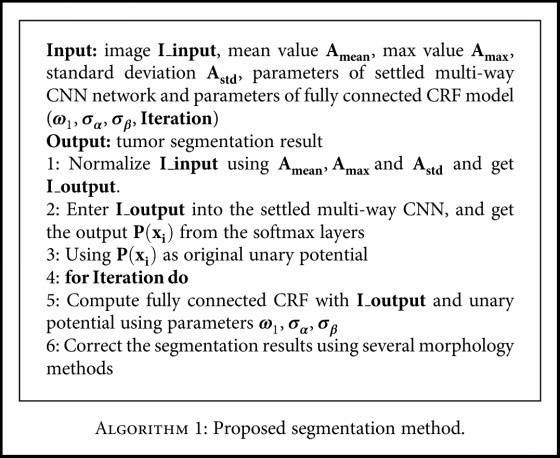
Algorithm 1: Proposed segmentation method.

**Table 1:** Patients characteristics.

Characteristics	Quantity	Percentage
Tumor grade		
Grade II	160	100.0%
Radiological diagnosis		
Astrocytoma	48	30.0%
Oligodendroglioma	25	15.6%
Oligodendrocytes astrocytoma	73	45.6%
Gender		
Male	81	50.6%
Female	79	49.4%
Category		
Training set	59	36.9%
Test set	101	63.1%

**Table 2:** Base line and improved CNN structure configurations.

CNN configuration			
Base line	Base line + deeper	Base line + more fc	Base line + deeper + more fc
Input			
Conv^1^3–64	Conv3–64	Conv3–64	Conv3–64
Conv3–64	Conv3–64	Conv3–64	Conv3–64
	Conv3–64		Conv3–64
Max-pool			
Conv3–128	Conv3–128	Conv3–128	Conv3–128
Conv3–128	Conv3–128	Conv3–128	Conv3–128
	Conv3–128		Conv3–128
Max-pool			
FC^2^–256		FC-4096	
FC-256		FC-4096	
FC-2			
Softmax			
Output			

^1^Conv: followed by the size of convolutional kernels and the number of filter banks. ^2^FC: followed by the number of fully connected layers.

**Table 3:** The optional parameters selected for the fully connected CRF (the first and the last digits represent the selected minimum and maximum values, and the middle number represents the chosen step size, expressed in MATLAB representation).

	*ω*_1_	*σ*_*α*_	*σ*_*β*_	Iterations
Selection range	5 : 5 : 10	20 : 5 : 70	3 : 1 : 10	5 : 1 : 10
The number of optional parameters	2	11	8	6
Optimized parameters	5	25	10	7

**Table 4:** Performance of different CNN structures on the training data.

CNN structure	DSC	PPV	Sensitivity	Total parameters
Base line	0.7609	0.6626	0.8937	1.9 × 10^6^
Base line + deeper	0.7872	0.6849	0.9253	2.1 × 10^6^
Base line + more fc	0.7678	0.6568	0.9239	4.3 × 10^7^
Base line + deeper +more fc	**0.8073**	**0.7296**	0.9036	4.3 × 10^7^

**Table 5:** Performance of different CNN structures on the test data.

CNN structure	DSC	PPV	Sensitivity
Base line	0.7834	0.6915	0.9034
Base line + deeper	0.7910	0.6987	0.9114
Base line + more fc	0.7841	0.6773	0.9306
Base line + deeper +more fc	**0.8021**	**0.7310**	0.8886

**Table 6:** Performance of different CNN structures associated with the CRF on the training data.

CNN structure	DSC	PPV	Sensitivity
One way CNN with CRF	0.8493	0.8355	0.8637
Early fusion with CRF	**0.8506**	0.8297	0.8745
Late fusion with CRF	0.8059	0.7415	**0.8825**

**Table 7:** Performance of different CNN structures associated with the CRF on the test data.

CNN structure	DSC	PPV	Sensitivity
One way CNN with CRF	0.8459	0.8577	0.8344
Early fusion with CRF	**0.8504**	0.8561	0.8447
Late fusion with CRF	0.8372	0.8113	**0.8649**
